# Dielectric light-trapping nanostructure for enhanced light absorption in organic solar cells

**DOI:** 10.1038/s41598-023-47898-9

**Published:** 2023-11-24

**Authors:** Seongcheol Ju, Hyeonwoo Kim, Hojae Kwak, Cheolhun Kang, Incheol Jung, Seunghyun Oh, Seung Gol Lee, Jeonghyun Kim, Hui Joon Park, Kyu-Tae Lee

**Affiliations:** 1 Department of Physics, Inha University, Incheon, 22212 Republic of Korea; 2 Department of Information and Communication Engineering, Inha University, Incheon, 22212 Republic of Korea; 3 Department of Electronic Convergence Engineering, Kwangwoon University, Seoul, 01897 Republic of Korea; 4 Department of Organic and Nano Engineering, Hanyang University, Seoul, 04763 Republic of Korea

**Keywords:** Optics and photonics, Optical materials and structures

## Abstract

Dielectric scatterers where Mie resonances can be excited in both electric and magnetic modes have emerged as a promising candidate for efficient light trapping (LT) in thin-film solar cells. We present that light absorption in organic solar cells (OSCs) can be significantly enhanced by a front-sided incorporation of a core–shell nanostructure consisting of a high-refractive-index dielectric nanosphere array conformally coated with a low-refractive-index dielectric layer. Strong forward light scattering of the all-dielectric LT structure enables the absorption in an organic semiconductor to be remarkably boosted over a broad range of wavelengths, which is attributed to interference of a simultaneous excitation of the electric and magnetic dipole resonant modes. The OSC with the LT structure shows the short-circuit current density (Jsc) of 28.23 mA/cm^2^, which is 10% higher than that of a flat OSC. We also explore how the LT structure affects scattering cross-sections, spectral multipole resonances, and far-field radiation patterns. The approach described in this work could offer the possibility for the improvement of characteristic performances of various applications, such as other thin-film solar cells, photodiodes, light-emitting diodes, and absorbers.

## Introduction

Organic solar cells (OSCs) have gained appreciable interest for their distinct benefits of achieving low cost, eco-friendliness, light weight, semitransparency, flexibility, and mass production^1–5^. Over the past few decades, a major amount of the studies on the OSCs has been devoted to the development of new material designs for photoactive layers and interfacial layers, morphology optimizations, transparent electrodes, and novel device configurations^6–18^. Extensive research efforts have been particularly focused on the development of innovative materials for the photoactive layer to markedly contribute to the power conversion efficiency (PCE) improvement in the OSCs. Recently, a new class of non-fullerene acceptor, Y6, which can match with a commercially available donor, PM6, is designed and successfully synthesized, and the Y6-based OSCs present much improved PCE of 18.85% with both conventional and inverted architectures^19–22^.

OSCs are generally based on the photoactive layer of a bulk heterojunction structure in which an electron donor and an electron acceptor are mixed. This is because of the difficulty to efficiently extract photogenerated charge carriers from the photoactive layer. Although the absorption in the photoactive layer of the OSCs increases with increasing a thickness of the photoactive layer, it is challenging to form a thick photoactive layer of the bulk heterojunction structure in the OSCs for the efficient photogenerated charge carrier extraction. The thickness of the photoactive layer in the OSCs is less than 100 nm, thus significantly limiting the PCE of the OSCs^23–26^. Such thin photoactive layers cannot harvest all photons in the solar spectrum, and hence light trapping (LT) has been key to improving the PCE of the OSCs. Various LT strategies based on localized surface plasmon resonances (LSPRs) in plasmonic nanoparticles, nanopyramids, nanorods, and core–shell nanostructures^27–36^, SPRs in metallic nanostructures^37–40^, photonic resonances in photonic crystal nanostructures^41,42^, and dielectric nanoparticles^43,44^ have been demonstrated. However, parasitic optical absorption losses in the metals are not negligible and the absorption improvement is limited to relatively narrow wavelength regions of the solar spectrum due to the excitation of the only electric resonance in the metallic nanostructures. Additional challenges arise in creating the LT nanostructures embossed in the photoactive layer, notably increasing defect densities, which can result in a significant decrease in the PCE of the OSCs. Therefore, there is a critical demand to develop a highly efficient and broadband LT structure that can provide performance enhancement of the OSCs.

Here we demonstrate that the LT structure comprising a high-refractive-index (HRI) dielectric nanosphere array (DNA) with a low-refractive-index (LRI) anti-reflective (AR) layer, which can be integrated on top of the OSCs. An efficient broadband LT effect is achieved by optimizing geometrical parameters of the HRI DNA/LRI AR structure, where the strong light scattering occurs in the forward direction and thus an optical path length in the photoactive layer is increased leading to the enhanced absorption over a broad wavelength range. This is ascribed to interference between electric and magnetic multipole resonances in the all-dielectric LT structure. After the incorporation of the LT structure, the short-circuit current density (Jsc) of the OSC with the 80 nm-thick PM6:Y6 photoactive layer is 28.23 mA/cm^2^, which is 10% higher than that of a planar OSC. Moreover, we investigate the effect of the LT structure on scattering cross-sections, multipole resonance contributions, and far-field radiation patterns. The presented scheme can be easily applied to improve the optical property of diverse applications, including other thin-film solar cells, sensors, photodetectors, and absorbers.

## Results and discussion

**Figure 1:**
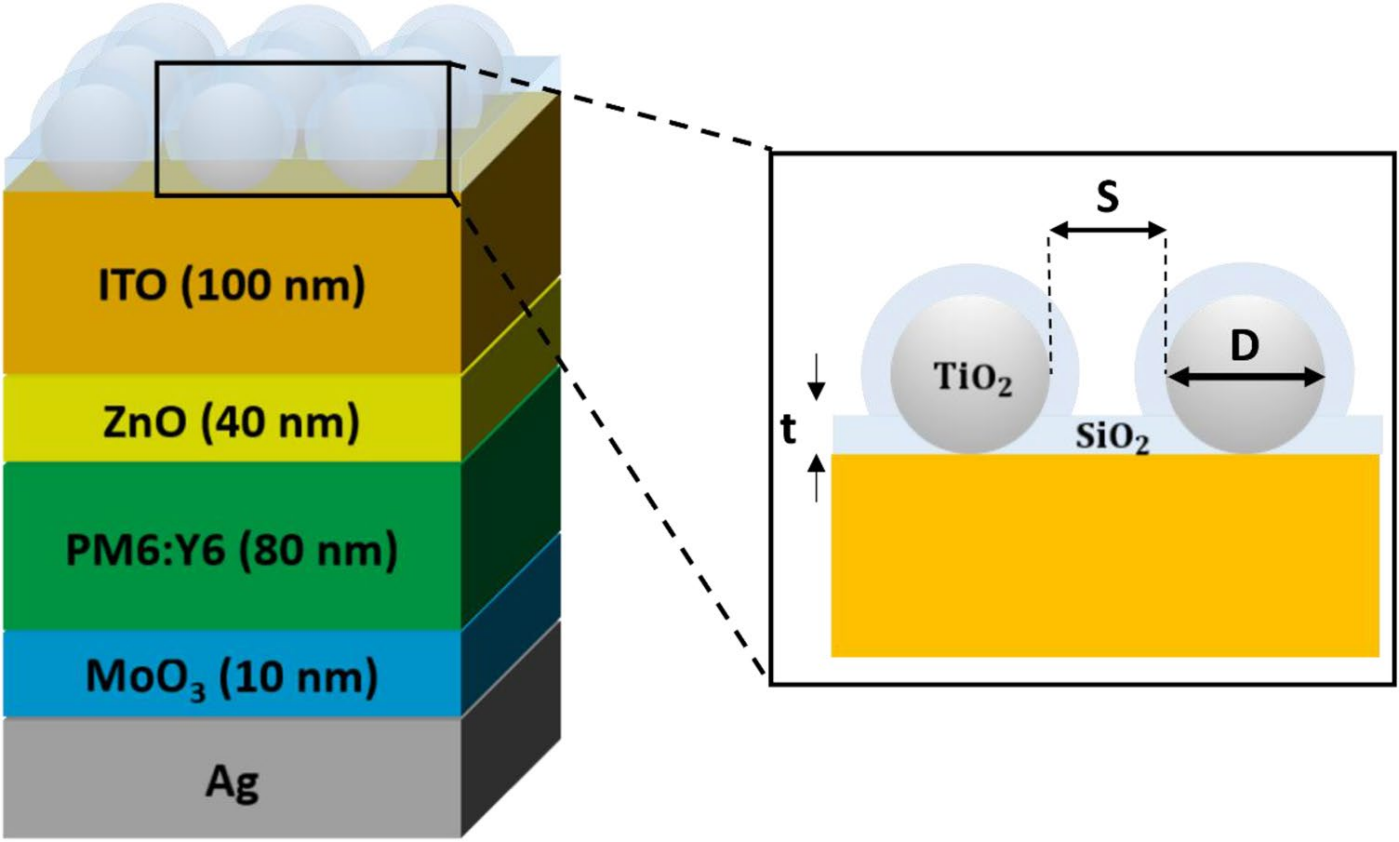
Schematic diagram of a flat OSC with an all-dielectric LT structure consisting of the HRI DNA conformally coated with the LRI AR coating.

In Fig. 1, a schematic view of a conventional flat OSC structure incorporated with the all-dielectric LT structure constructed from the HRI DNA conformally coated with the LRI AR layer is depicted. The OSC structure comprises 100 nm-thick indium tin oxide (ITO) as a top transparent electrode, 40 nm-thick zinc oxide (ZnO) as an electron-transporting layer, 80 nm-thick PM6:Y6 as a bulk heterojunction photoactive layer, 10 nm-thick molybdenum trioxide (MoO_3_) as a hole-transporting layer, and 100 nm-thick silver (Ag) as a bottom electrode and a thick mirror. Although exploring alternative materials for the buffer layers and finely tuning the thickness of the related layers is important and required for achieving the optimized solar cell performance, the optimized interfacial layers are primarily determined by the experiment from the electrical aspect (e.g., energy band alignment, etc.). Thus, we selected ZnO as the electron transporting layer and MoO3 as the hole transporting layer, both of which are the interfacial layers most commonly used in the OSC with the PM6:Y6 photoactive layer^45^. Since the thicknesses of the interfacial layers can significantly impact the electrical properties of the solar cell, it is important to clarify that our primary focus in this study is not on the electrical characteristics but rather on optical properties. This focus makes it challenging to independently evaluate the impact of transport layer thickness on electrical properties within the scope of this paper. The LT structure can be formed at the final step of a device fabrication and can be used as an excellent encapsulation layer, thus greatly reducing charge carrier recombination. The absorption in the photoactive layer of the OSCs can be significantly enhanced by exploiting the LSPRs in the plasmonic LT structure. However, the metals show non-trivial parasitic absorption losses and the absorption enhancement occurs in the limited wavelength regimes of the solar spectrum, which is attributed to the fact that the only electric resonance can be excited in the plasmonic LT structures^46–48^. Additionally, defect densities can be notably increased by embossing the LT nanostructures in the photoactive layer, thus causing the PCE of the OSCs to be significantly reduced. As the HRI dielectric with transparency in the visible wavelength region is required to achieve the strong light scattering efficiency in the forward direction while minimizing the parasitic optical absorption losses in the LT structure, titanium dioxide (TiO_2_) is chosen. Silicon dioxide (SiO_2_) is selected for the LRI AR layer. We note that a self-assembly can be used to form the front-located HRI DNA, and either chemical vapor deposition or atomic layer deposition can be used to deposit the conformal LRI AR coating layer. We note that an array of particles is constructed in a hexagonal arrangement utilizing the self-assembly although a square array structure is selected in our study^49–51^. Calculated absorption spectra in the photoactive layer obtained from the hexagonal and square array structures with the same geometrical parameters are provided in Supporting Information (Fig. S1). The overall shape of the spectra attained from the hexagonal and square structures remains almost similar with a trivial discrepancy, which would be attributed to the difference in the index matching condition resulting from the structural configuration.

Optimal geometrical parameters including a diameter (*D*) and a spacing (*S*) of the HRI DNA, and a thickness (*t*) of the LRI AR layer are obtained by performing the three-dimensional (3D) simulation using a commercial software based on the finite element method (COMSOL Multiphysics). The optimized parameters of the LT structure allow the scattering to be strong in the forward direction so that an optical path length in the photoactive layer can be extended and hence the absorption in the photoactive layer can be enhanced. Optical constant of PM6:Y6 is obtained from a previous work^52^ and the optical constants of Ag, MoO_3_, ZnO, ITO, TiO_2_, and SiO_2_ are obtained from a website (https://refractiveindex.info), which are provided in Supporting Information (Fig. S2).

**Figure 2:**
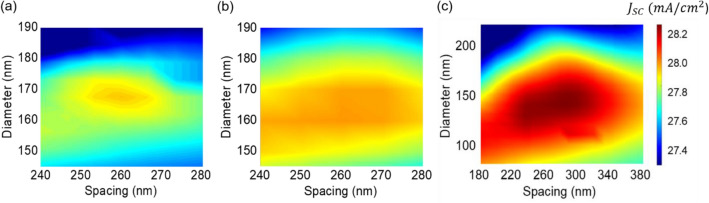
2D contour plots of the Jsc as a function of *S* and *D* of the HRI DNA with the conformal LRI AR coating thickness of (**a**) 0 nm, (**b**) 25 nm, and (**c**) 50 nm.

The performance of the LT structure can be evaluated by investigating the short-circuit current density (Jsc) associated with the absorption in the photoactive layer of the OSCs. By optimizing the featured parameters of the LT structure via a parameter sweep, the optical path length is increased thus maximizing the Jsc. The Jsc can be computed by the following equation:

1 $${J}_{SC}={\int }_{300 nm}^{1000 nm}\frac{e\lambda }{ch}QE\left(\lambda \right){I}_{AM1.5}\left(\lambda \right)d\lambda$$

where *h*, *c*, *e*, *λ*, and I_AM1.5_(λ) are Planck’s constant, the velocity of light in free space, electron charge, wavelength, and spectral irradiance of the standard AM 1.5 spectrum, respectively. We note that the quantum efficiency (QE(λ)) is equal to the absorption spectrum in the photoactive layer assuming that all the photons absorbed in the photoactive layer can contribute to the photocurrent generation without any recombination loss. Figure 2a–c present simulated 2D contour plots of the Jsc as a function of *D* and *S* of the HRI DNA with the fixed *t* of the LRI AR coating of 0, 25, and 50 nm, respectively. Without the LRI AR coating shown in Fig. 2a, a maximum Jsc of 27.20 mA/cm^2^ occurs at *D* = 180 nm and *S* = 260 nm, with an enhancement of 5.9% as compared to that of the planar OSC structure (25.68 mA/cm^2^). With the LRI AR coating illustrated in Fig. 2b and c, the maximum Jsc values of 28.09 mA/cm^2^ at *D* = 160 nm and *S* = 260 nm, and 28.25 mA/cm^2^ at *D* = 140 nm and *S* = 280 nm are attained when the thicknesses of the LRI AR coating are 25 and 50 nm, respectively. As can be seen from the figures, it is apparent that the Jsc values are nearly insensitive with respect to *S* of the HRI DNA without and with the LRI AR coating. Based on these findings, we fix the spacing, as it has been observed to be wide enough to neglect interparticle interactions and to be independent of the optimal values for the short-circuit current density across all SiO_2_ coating thicknesses. Consequently, the diameter and coating layer thickness were chosen as the variables for optimization. It is also found that the Jsc gets reduced when *t* is thicker than 50 nm from the calculation. These suggest that *t* of the LRI AR coating can be swept to find the optimal *t* leading to the maximum Jsc at the fixed *S* = 260 nm.

**Figure 3:**
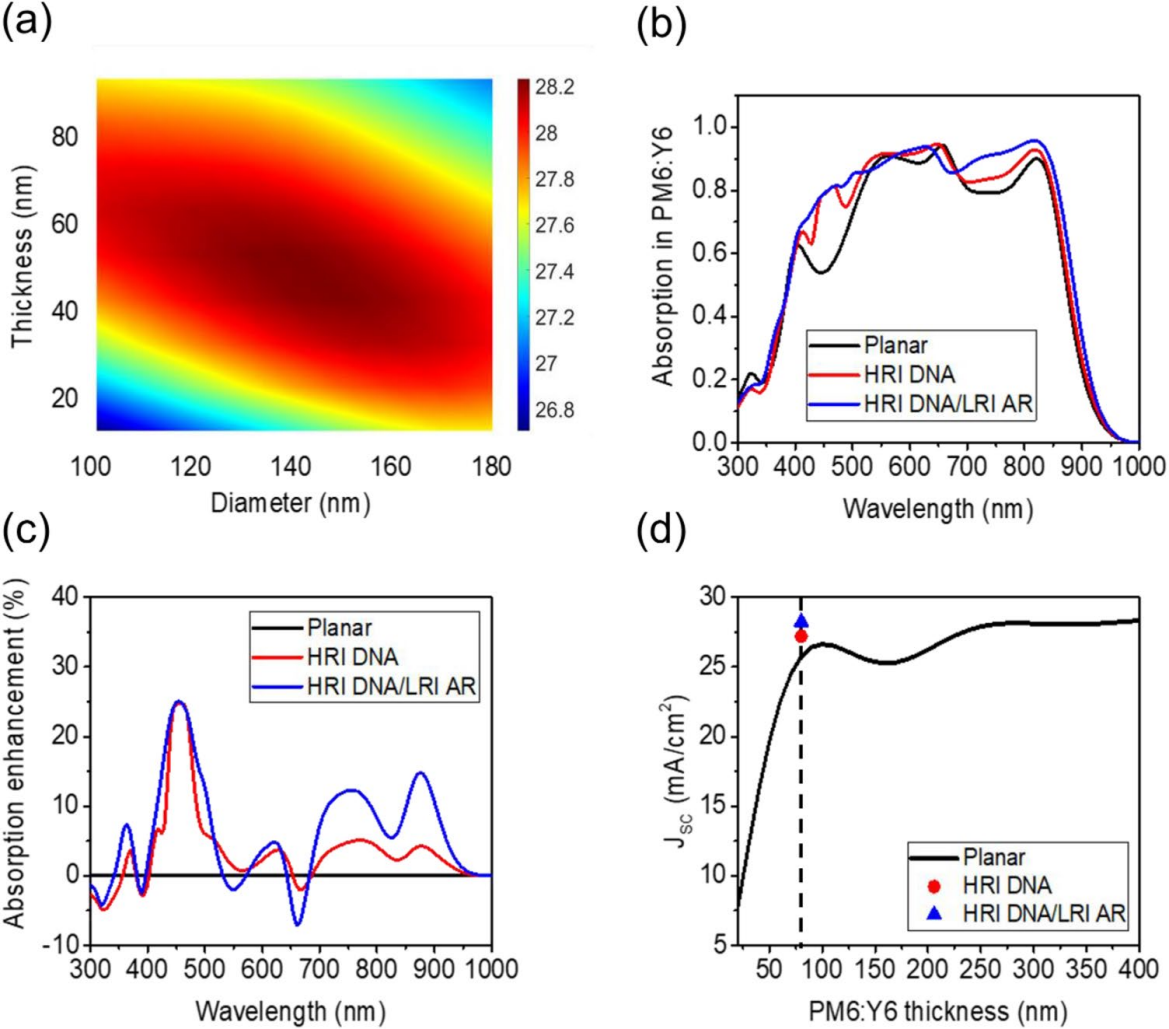
(**a**) Simulated 2D contour plot of the Jsc as a function of *D* of the HRI DNA and *t* of the LRI AR coating with *S* of the HRI DNA fixed at 260 nm. (**b**) Calculated absorption spectra and (**c**) absorption enhancement in the PM6:Y6 photoactive layer of the planar OSC structure (black), with the HRI DNA (red), and the HRI DNA/LRI AR (blue). (**d**) Jsc of the planar OSC as a function of the thickness of the PM6:Y6 photoactive layer (black) plotted together with the Jsc of the planar OSC integrated with the HRI DNA (red circle) and the HRI DNA/LRI AR layer (blue triangle).

The 2D contour plot of the Jsc as a function of *D* of the HRI DNA and *t* of the LRI AR coating with *S* fixed at 260 nm is exhibited in Fig. 3a, showing that the maximum Jsc of 28.23 mA/cm^2^ can be achieved from the LT structure with *D* = 150 nm, *S* = 260 nm and *t* = 40 nm. Figure 3b depicts calculated absorption spectra in the PM6:Y6 photoactive layer of the planar OSC (black) and the planar OSC incorporated with the HRI DNA (red) and the HRI DNA/LRI AR coating (blue). There are four peaks in the absorption spectrum of the flat OSC at 400, 550, 650, and 820 nm, all of which are ascribed to the Fabry–Perot resonance. There are also two distinct dips at 450 nm and 750 nm between the peaks causing a significant decrease in the PCE of the OSCs. The integration of the HRI DNA/LRI AR with the flat OSC structure enables the light scattering to occur strongly in the forward direction, thereby increasing the optical path length in the PM6:Y6 photoactive layer. Thus, the enhanced absorption can be accomplished over a wide range of wavelength ranges, yielding the improved Jsc. Such a broadband absorption enhancement arises from the simultaneous excitation of both electric and magnetic resonances in the HRI DNA/LRI AR, which will be explored later.

As is seen from Fig. 3b, the OSC integrated with the HRI DNA at *D* = 180 nm and *S* = 260 nm shows the enhanced absorption in PM6:Y6 at 450 nm, displaying the Jsc of 27.20 mA/cm^2^, which is the 5.9% improvement as compared to that of the planar OSC (25.68 mA/cm^2^). The further absorption enhancement in PM6:Y6 at the longer wavelength ranges between 700 and 900 nm can be achieved by overlaying the HRI DNA with the optimal LRI AR coating, presenting the Jsc of 28.23 mA/cm^2^ with the improvement of 10% as compared to the flat OSC. The absorption enhancement after introducing the HRI DNA and the HRI DNA/LRI AR coating into the OSC structure is revealed in Fig. 3c. It is found that the enhancement in the Jsc in the wavelength range between 300 and 400 nm is insignificant, which is because the absorption in the constituent layers is not negligible in this wavelength range (see the extinction coefficient of the materials in Fig. S2). The absorption spectra in each layer of the OSC with the HRI DNA/LRI AR coating are provided in Supporting Information (Fig. S3), displaying that the absorption occurs in all the layers except SiO_2_ for the ultraviolet (UV) wavelength range from 300 to 400 nm. Since the stability of the OSCs is significantly affected by the long-standing UV irradiation, the absorption for the wavelength range between 300 and 400 nm is certainly helpful in improving the stability of the OSCs. To investigate the AR effect, the SiO_2_ part that connects directly with ITO in the cell is replaced by TiO_2_ to see if the effective refractive index decreases. Calculated absorption spectra in the photoactive layer obtained from the proposed structures with 20 nm-thick and 40 nm-thick TiO_2_ thin films on top of ITO are given in Supporting Information (Fig. S4). As can be seen from the figure, the anti-reflection effect over a broad range of wavelengths is reduced by introducing the TiO_2_ thin film, which would be attributed to the weakened Mie resonance effect. In Fig. 3d, the calculated Jsc of the planar OSC as a function of the thickness of the PM6:Y6 photoactive layer is described. Although the Jsc value increases with increasing the thickness of PM6:Y6, the Jsc becomes saturated at around 26 mA/cm^2^, and the maximum Jsc of 26.63 mA/cm^2^ can be attained when the thickness of PM6:Y6 is 100 nm from the planar OSC. This suggests that the Jsc of the planar OSC is limited to the maximum value of 26.63 mA/cm^2^ even with increasing the thickness of PM6:Y6 so the LT effect is highly necessary to enhance the absorption in PM6:Y6. The Jsc of the OSC with the 80 nm-thick photoactive layer incorporated with the HRI DNA is 27.2 mA/cm^2^, which is 5.9% higher than that of the planar OSC. This is comparable with the Jsc of the planar OSC with the 230 nm-thick photoactive layer. The integration of the HRI DNA/LRI AR with the OSC with the 80 nm-thick photoactive layer leads to the Jsc of 28.23 mA/cm^2^ that is 10% higher than that of the flat OSC and analogous to the Jsc of the flat OSC with the 390 nm-thick photoactive layer. While this study is primarily focused on the optical properties of the OSC, the PCE of the OSC integrated with the HRI DNA/LRI AR can be evaluated by taking Voc = 0.86 V and FF = 73.2% in a prior work resulting in 15.3% of the PCE^19^.

**Figure 4:**
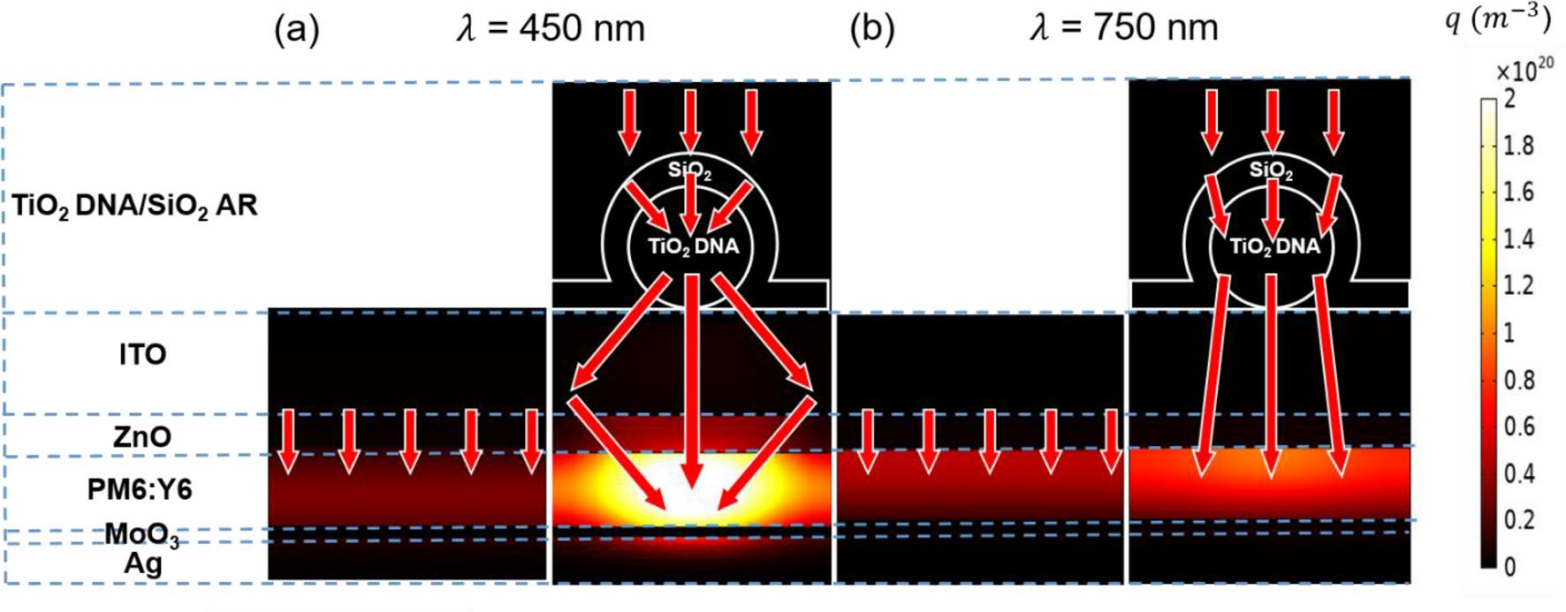
Absorption distribution profiles in the planar OSC structures without (left) and with (right) the HRI DNA/LRI AR at (**a**) λ = 450 nm and (**b**) λ = 750 nm. Red arrows represent the Poynting vectors.

In Fig. 4, absorption distributions into the OSC structure and Poynting vectors without and with the HRI DNA/LRI AR LT structure at the wavelengths of 450 nm and 750 nm where the significant absorption enhancement is achieved are displayed. The power absorbed per unit volume in each layer is given by ${P}_{abs}=\frac{1}{2}\omega {\varepsilon }^{{\prime}{\prime}}{\left|E\right|}^{2}$ where ω is the angular frequency, ${\varepsilon }^{{\prime}{\prime}}$ is the imaginary part of the dielectric permittivity, and ${\left|E\right|}^{2}$ is the electric field intensity. The absorption density (${p}_{abs}$) can be obtained by normalizing ${P}_{abs}$ by the light source, which is employed to compute the absorption at a certain wavelength by performing a volume integral of the ${p}_{abs}$ ($Absorption\left(\lambda \right)=\int {p}_{abs}(\lambda )dV$). The greatly enhanced absorption in PM6:Y6 at 450 nm is achieved from the OSC integrated with the HRI DNA/LRI AR as compared with the flat OSC as exhibited in Fig. 4a. As is seen from the figure, the intense electric field is formed in PM6:Y6 at 450 nm since the HRI DNA/LRI AR acts as nano-lenses, which is highly beneficial to the OSC with the thin photoactive layer. This better coupling of light into PM6:Y6 is attributed to the strong light scattering of the HRI DNA/LRI AR in the forward direction, which is enabled by interference between the electric dipole and the magnetic dipole. The focusing effect can also be found by exploring the Poynting vector describing a direction of the energy flow as indicated by red arrows in the figure. The improved absorption in PM6:Y6 observed at 750 nm is due to the strong far-field light scattering of the HRI DNA/LRI AR, thus allowing the orientation of vertically incident light to be diverted toward more horizontal directions. This increases the optical path length in PM6:Y6 as is seen from the Poynting vector in Fig. 4b. Moreover, the AR effect arising from a better matching of the refractive index and a graded index profile comprising TiO_2_ covered by SiO_2_ is responsible for increasing the absolute amount of light reaching PM6:Y6. The theoretically optimized refractive index of the AR coating between air and TiO_2_ at 750 nm is $\sqrt{1\times 2.35}=1.53$ that matches well with the refractive index of SiO_2_ that is 1.48, suggesting that SiO_2_ is a suitable candidate for the AR. It is important to note that the conformal LRI AR coating redirects normally incident light toward the HRI DNA, hence increasing the scattering cross-section of the HRI DNA as observed from the Poynting vector flow in Fig. 4a and b. It is also worth noting that exploring optimized geometry or refractive index combination of the core–shell LT structure can further improve the LT effect.

**Figure 5:**
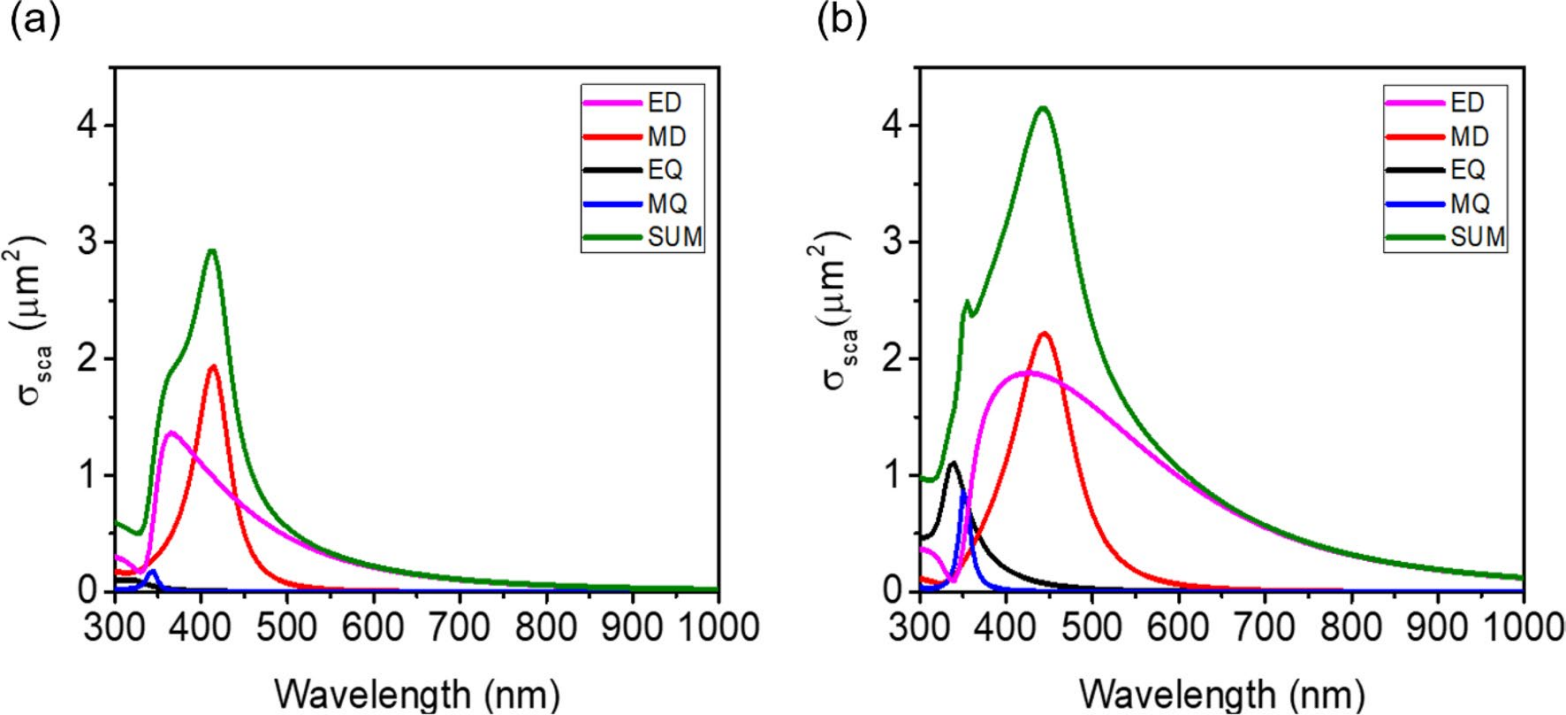
Scattering cross-sections (σ_sca_) and the corresponding multipole decomposition for (**a**) the HRI DN with *D* of 180 nm in air and (**b**) the HRI DN/LRI AR with *D* of 150 nm and *t* of 40 nm in air.

In Fig. 5a and b, scattering cross-section (*σ*_*sca*_) spectra and the corresponding multipoles’ contributions for the HRI DN with *D* of 180 nm in air and for the HRI DN/LRI AR with *D* of 150 nm and *t* of 40 nm in air are presented. We note that the scattering property of a freestanding configuration of the nanoparticle is investigated in our study, but the scattering cross-section gets broadened with invariant resonance positions in multipole decomposition with a substrate^53^. According to the Mie theory, more forward light scattering occurs when the size of the nanoparticle is increased. The overall size of the DN is increased by overlaying the HRI DN with the LRI AR, thus producing the strong forward scattering. Additionally, as shown from the Poynting vector flow in Fig. 4a and b, the σ_sca_ of the HRI DN/LRI AR increases by bending incident light toward the HRI DN with the help of the conformal LRI AR layer. Increasing the size and the spacing of the nanoparticles enables peaks of the electric and magnetic resonances to be shifted to the longer wavelength regime^54–56^. Moreover, broadening and shifting toward the longer wavelength range occur for the electric resonance while broadening but a relatively trivial shift exists for the magnetic resonance when the nanoparticles are encircled by any materials according to the Mie scattering theory.

**Figure 6:**
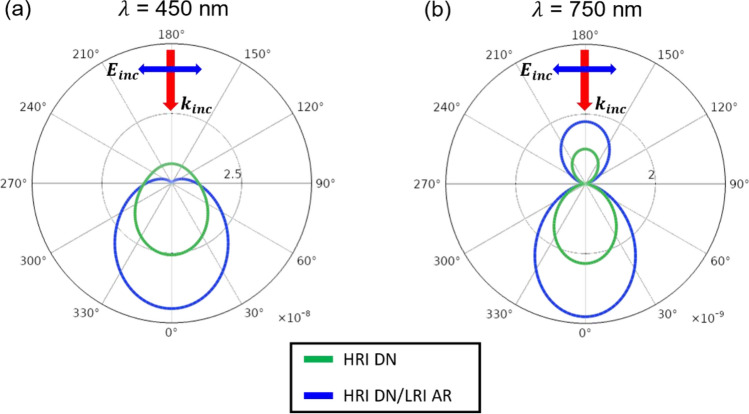
Radiation patterns of the HRI DN with *D* of 180 nm in air (green) and the HRI DN/LRI AR with *D* of 150 nm and *t* of 40 nm in air (blue) at (**a**) λ = 450 nm and (**b**) λ = 750 nm.

The electric dipole (ED) and the magnetic dipole (MD) resonances of the HRI DN show a peak at 365 nm and 415 nm, respectively, while the contribution of the electric quadrupole (EQ) and the magnetic quadrupole (MQ) to the total *σ*_*sca*_ is insignificant as exhibited in Fig. 5a. The ED and the MD are balanced at 395 nm, known as the first Kerker condition, presenting nearly-zero light scattering in the backward direction^57^. This implies that the only forward scattering occurs, thus significantly improving the absorption in PM6:Y6 and hence the enhanced Jsc. As depicted in Fig. 3b, the first dip in the absorption spectrum in PM6:Y6 of the planar OSC appears at 450 nm that is in the vicinity of 395 nm where the ED and the MD resonances are spectrally overlapped leading to the strong light scattering in the forward direction. Although the remarkable enhancement of the absorption in PM6:Y6 is achieved at 450 nm after placing the HRI DN on top of the OSC, the total σsca is small at 750 nm where the second dip in the absorption spectrum in PM6:Y6 exists. Thus, the improvement of the absorption at 750 nm with the integration of the HRI DN into the flat OSC is trivial. As the ED is sensitive to the surrounding medium, the optimized LRI AR layer conformally coated on the HRI DN allows the ED resonance to be moved toward the longer wavelength with a broadened profile. By covering the HRI DN with the LRI AR with the optimal thickness, the wavelength where the ED and the MD resonances are matched resulting in the strong forward scattering appears at 425 nm and 465 nm that are much close to the first dip in the absorption in PM6:Y6 (450 nm). Besides, the ED is broadened with the improved efficiency after adding the LRI AR coating so that the total σsca is greatly enhanced over the longer wavelength region ranging from 700 to 1000 nm. This contributes to the significant improvement in the absorption in PM6:Y6 at the wavelengths between 700 and 850 nm. The total σsca is calculated using the sum of the contributions from the different multipole moments. The scattering cross-sections for the ED (${\sigma }_{ED}$),), MD (${\sigma }_{MD}$), EQ (${\sigma }_{EQ}$), and MQ (${\sigma }_{MQ}$) are computed by using the following equations^58,59^:

2 $$\begin{aligned} \sigma_{sca} & = \sigma_{ED} + \sigma_{MD} + \sigma_{EQ} + \sigma_{MQ} + \cdots \\ & = \frac{{k_{0}^{4} }}{{6\pi \varepsilon_{0}^{2} E_{0}^{2} }}\left[ {\mathop \sum \limits_{\alpha } \left( {\left| {p_{\alpha } } \right|^{2} + \frac{{\left| {m_{\alpha } } \right|^{2} }}{c}} \right) + \frac{1}{120}\mathop \sum \limits_{\alpha \beta } \left( {\left| {kQ_{\alpha \beta } } \right|^{2} + \left| {\frac{{kM_{\alpha \beta } }}{c}} \right|^{2} } \right) + \cdots } \right] \\ \end{aligned}$$

3 $$\sigma_{ED} = \frac{{k_{0}^{4} }}{{6\pi \varepsilon_{0}^{2} E_{0}^{2} }}\mathop \sum \limits_{\alpha } \left| {p_{\alpha } } \right|^{2}$$

4 $$\sigma_{MD} = \frac{{k_{0}^{4} \varepsilon \mu_{0} }}{{6\pi \varepsilon_{0} E_{0}^{2} }}\mathop \sum \limits_{\alpha } \left| {m_{\alpha } } \right|^{2}$$

5 $$\sigma_{EQ} = \frac{{k_{0}^{6} \varepsilon }}{{720\pi \varepsilon_{0}^{2} E_{0}^{2} }}\mathop \sum \limits_{\alpha \beta } \left| {Q_{\alpha \beta } } \right|^{2}$$

6 $$\sigma_{MQ} = \frac{{k_{0}^{6} \varepsilon^{2} \mu_{0} }}{{80\pi \varepsilon_{0} E_{0}^{2} }}\mathop \sum \limits_{\alpha \beta } \left| {M_{\alpha \beta } } \right|^{2}$$

where ${\mathrm{p}}_{{\alpha }}=-\frac{1}{\mathrm{i}\omega }\left\{\int {\mathrm{d}}^{3}{\mathrm{rJ}}_{{\alpha }}^{\upomega }{\mathrm{j}}_{0}\left(\mathrm{kr}\right)+\frac{{\mathrm{k}}^{2}}{2}\int {\mathrm{d}}^{3}\mathrm{r}\left[3\left(\mathrm{r}\cdot {\mathrm{J}}_{\upomega }\right){\mathrm{r}}_{{\alpha }}-{\mathrm{r}}^{2}{\mathrm{J}}_{{\alpha }}^{\upomega }\right]\frac{{\mathrm{j}}_{2}(\mathrm{kr})}{{\left(\mathrm{kr}\right)}^{2}}\right\}$ ,${\mathrm{m}}_{{\alpha }}=\frac{3}{2}\int {\mathrm{d}}^{3}\mathrm{r}{\left(\mathrm{r}\times {\mathrm{J}}_{\upomega }\right)}_{{\alpha }}\frac{{\mathrm{j}}_{1}(\mathrm{kr})}{\mathrm{kr}}$, ${\mathrm{Q}}_{{\alpha \beta }}=-\frac{3}{\mathrm{i\omega }}\left\{\int {\mathrm{d}}^{3}\mathrm{r}[{({\mathrm{r}}_{\upbeta }\mathrm{J}}_{{\alpha }}^{\upomega }+{{\mathrm{r}}_{{\alpha }}\mathrm{J}}_{\upbeta }^{\upomega })-2\left(\mathrm{r}\cdot {\mathrm{J}}_{\upomega }\right){\updelta }_{{\alpha \beta }}]\frac{{\mathrm{j}}_{1}\left(\mathrm{kr}\right)}{\mathrm{kr}}+2{\mathrm{k}}^{2}\int {\mathrm{d}}^{3}\mathrm{r}\left[5{\mathrm{r}}_{{\alpha }}{\mathrm{r}}_{\upbeta }\left(\mathrm{r}\cdot {\mathrm{J}}_{\upomega }\right)-\left({\mathrm{r}}_{{\alpha }}{\mathrm{J}}_{\upbeta }+{\mathrm{r}}_{\upbeta }{\mathrm{J}}_{{\alpha }}\right){\mathrm{r}}^{2}-{\mathrm{r}}^{2}\left(\mathrm{r}\cdot {\mathrm{J}}_{\upomega }\right){\updelta }_{{\alpha \beta }}\right]\frac{{\mathrm{j}}_{3}(\mathrm{kr})}{{\left(\mathrm{kr}\right)}^{3}}\right\}$, and ${\mathrm{M}}_{{\alpha \beta }}=15\int {\mathrm{d}}^{3}\mathrm{r}\{{{\mathrm{r}}_{{\alpha }}\left(\mathrm{r}\times {\mathrm{J}}_{\upomega }\right)}_{\upbeta } +{{\mathrm{r}}_{\upbeta }\left(\mathrm{r}\times {\mathrm{J}}_{\upomega }\right)}_{{\alpha }}\}\frac{{\mathrm{j}}_{2}(\mathrm{kr})}{{\left(\mathrm{kr}\right)}^{2}}$ represent the multipole momentums, E_0_ is the electric field amplitude of the incident plane wave, *c* is the speed of light, *k* is the wavenumber in the material and *k*_0_ is the wavenumber in vacuum. *α, β* refer to *x*, *y*, *z*. *J*_*w*_ is the induced current density and can be obtained from this equation (*J*_*ω*_ = *iωε*_0_ (*ε*_*r*_* − *1) *E*_*ω*_(*r*)), *E*_*ω*_(*r*) is the electric field distribution, *ε*_*r*_ is the relative permittivity of the TiO_2_ sphere, and *ε*_0_ is the permittivity of vacuum. Finally, J_*i*_ is the expression of the *i*-th order spherical Bessel function. It is worth noting that the use of exact multipole moments is valid for all particle sizes, and all physical quantities obtained through the equations will be exact. The third family of the multipoles is ignored in this study. Previous work has shown that the third family of multipoles, the toroidal multipole moments, is just a high-order terms via an expansion of the electric multipole moments^60^.Far-field radiation patterns of the HRI DN and the HRI DN/LRI AR at the wavelength of 450 nm and 750 nm are provided in Fig. 6a and b. A directive far-field radiation pattern with a small backward scattering occurs for the HRI DN with *D* of 180 nm with at the wavelength of 450 nm, whereas the backward scattering becomes zero due to completely balanced ED and MD moments satisfying the first Kerker condition for the HRI DN/LRI AR with *D* of 150 nm and *t* of 40 nm at the wavelength of 450 nm as shown in Fig. 6a. In addition to the zero backscattering, the forward-scattering intensity of the HRI DN/LRI AR is 1.8 times higher than that of the HRI DN, with a larger scattering angle, thus allowing the optical path length in PM6:Y6 to be significantly prolonged yielding much improved Jsc. Although the backscattering appears at the wavelength of 750 nm as exhibited in Fig. 6b, the scattering occurs strongly in the forward direction for both the HRI DN and the HRI DN/LRI AR. We note that the far-field intensity at the wavelength of 450 nm is about 10 times greater than that at the wavelength of 750 nm.

## Conclusion

In summary, we have investigated a novel front-sided, all-dielectric LT structure consisting of the HRI DNA conformally coated by the LRI AR with the significant absorption enhancement in the photoactive layer of the OSCs. By optimizing the geometrical parameters of the HRI DNA/LRI AR, the enhanced forward-scattering occurs over a broad range of wavelengths leading to the increase in the optical path length in the photoactive layer. This is enabled by exploiting strong far-field forward-scattering resulting from the dielectric nanosphere with high index of refraction in combination with the efficient AR effect achieved by the better index matching and the graded refractive index profile. The maximum Jsc of 28.23 mA/cm^2^ is attained from the HRI DNA/LRI AR with *D* = 150 nm, *S* = 260 nm and *t* = 40 nm, which is 10% higher than that of the planar OSC structure. The approach described in this work may pave the way for a variety of applications, such as other solar cells, photosensors, and absorbers.

## Method

The three-dimensional finite element method (FEM) based simulations were conducted using a commercial software (COMSOL Multiphysics). Maxwell's equations were solved for each discretized element in a mesh. Periodic boundary conditions were applied in the (*x*, *y*) plane for a single nanosphere structure and a perfect matching layer (PML) was implemented at the topmost and bottommost layers of the solar cell to mitigate reflections at the open boundary. The mesh thickness for each layer was set to 1/3 of the minimum value of the geometry parameters to ensure accurate representation. The short-circuit current density (Jsc) was calculated by using Eq. (1) and the AM1.5 spectrum ranging from 300 to 1000 nm was considered in the calculation. By applying an electric field to the free-standing TiO_2_/SiO_2_ core–shell nanostructure, the total scattering cross-section spectra and the multipole decomposition spectra were computed using Eqs. (2)–(6).

### Supplementary Information

Supplementary Figures.

## Data Availability

The datasets used and/or analyzed during the current study available from the corresponding author on reasonable request.
