# Supplementary material for: Dielectric light-trapping nanostructure for enhanced light absorption in organic solar cells
Source: Sci Rep. 2023 Nov 24;13:20649. doi: 10.1038/s41598-023-47898-9 (PMC10673921; doi:10.1038/s41598-023-47898-9)
Supplement: Supplementary file 1 — Supplementary Figures. [file 41598_2023_47898_MOESM1_ESM.pdf]

Supplementary Information

Dielectric light-trapping nanostructure for enhanced light absorption in organic solar cells

Seongcheol Ju[1], Hyeonwoo Kim[1], Hojae Kwak[1], Cheolhun Kang[1], Incheol Jung[1], Seunghyun Oh[1], Seung Gol Lee[2], Jeonghyun Kim[3,*], Hui Joon Park[4,*], and Kyu-Tae Lee[1,*]

* Corresponding author.

[1]Department of Physics, Inha University, Incheon 22212, South Korea

[2]Department of Information and Communication Engineering, Inha University, 22212, Incheon, Republic of Korea

[3]Department of Electronic Convergence Engineering, Kwangwoon University, Seoul 01897, Republic of Korea

[4]Department of Organic and Nano Engineering, Hanyang University, Seoul 04763, Republic of Korea

* Prof. Kyu-Tae Lee (ktlee@inha.ac.kr)

* Prof. Hui Joon Park (huijoon@hanyang.ac.kr)

* Prof. Jeonghyun Kim (jkim@kw.ac.kr)

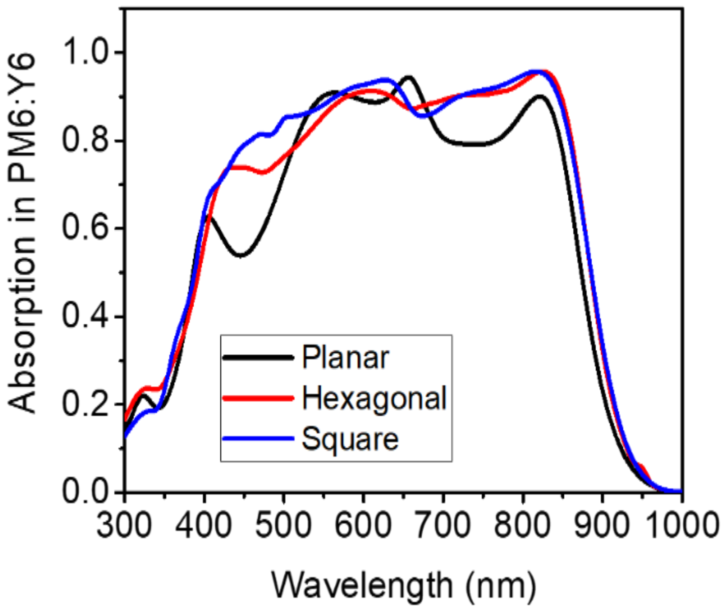

Figure S1. Calculated absorption spectra in the photoactive layer from planar, hexagonal array, and square array structures.

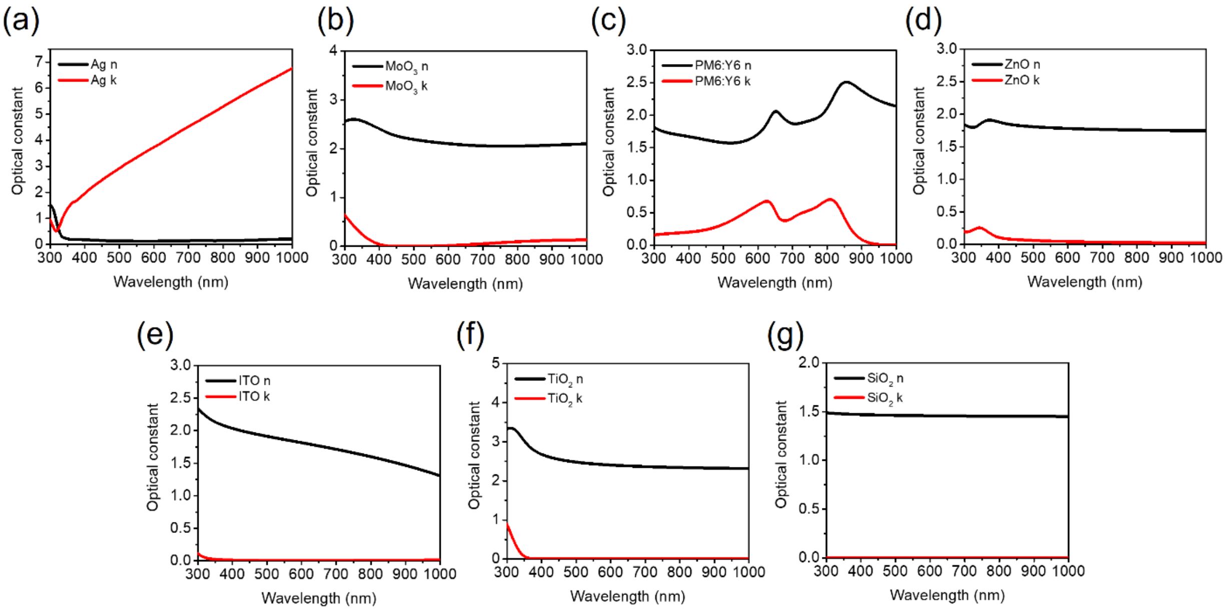

Figure S2. Spectra of optical constants for (a) Ag, (b) MoO_3, (c) PM6:Y6, (d) ZnO, (e) ITO, (f) TiO_2[1], and (g) SiO_2[2].

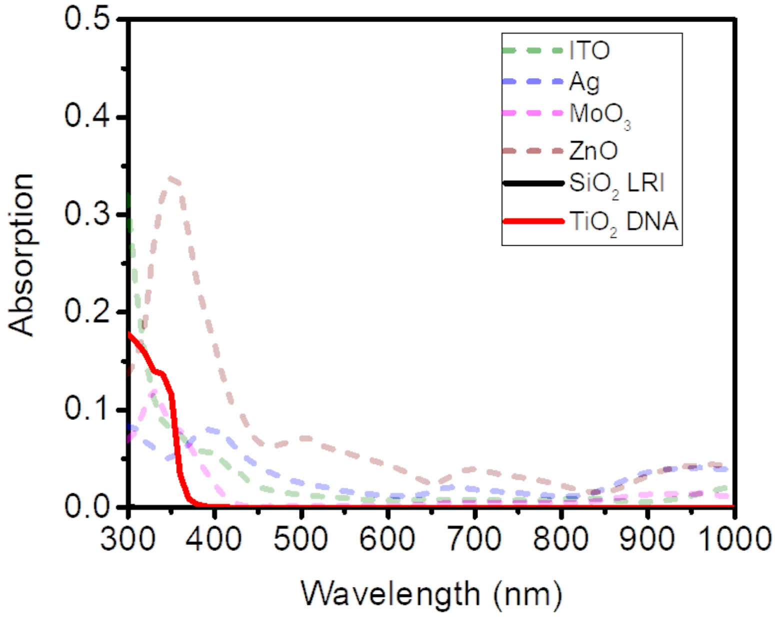

Figure S3. Calculated absorption spectra in each layer of the OSC with the HRI DNA/LRI AR coating.

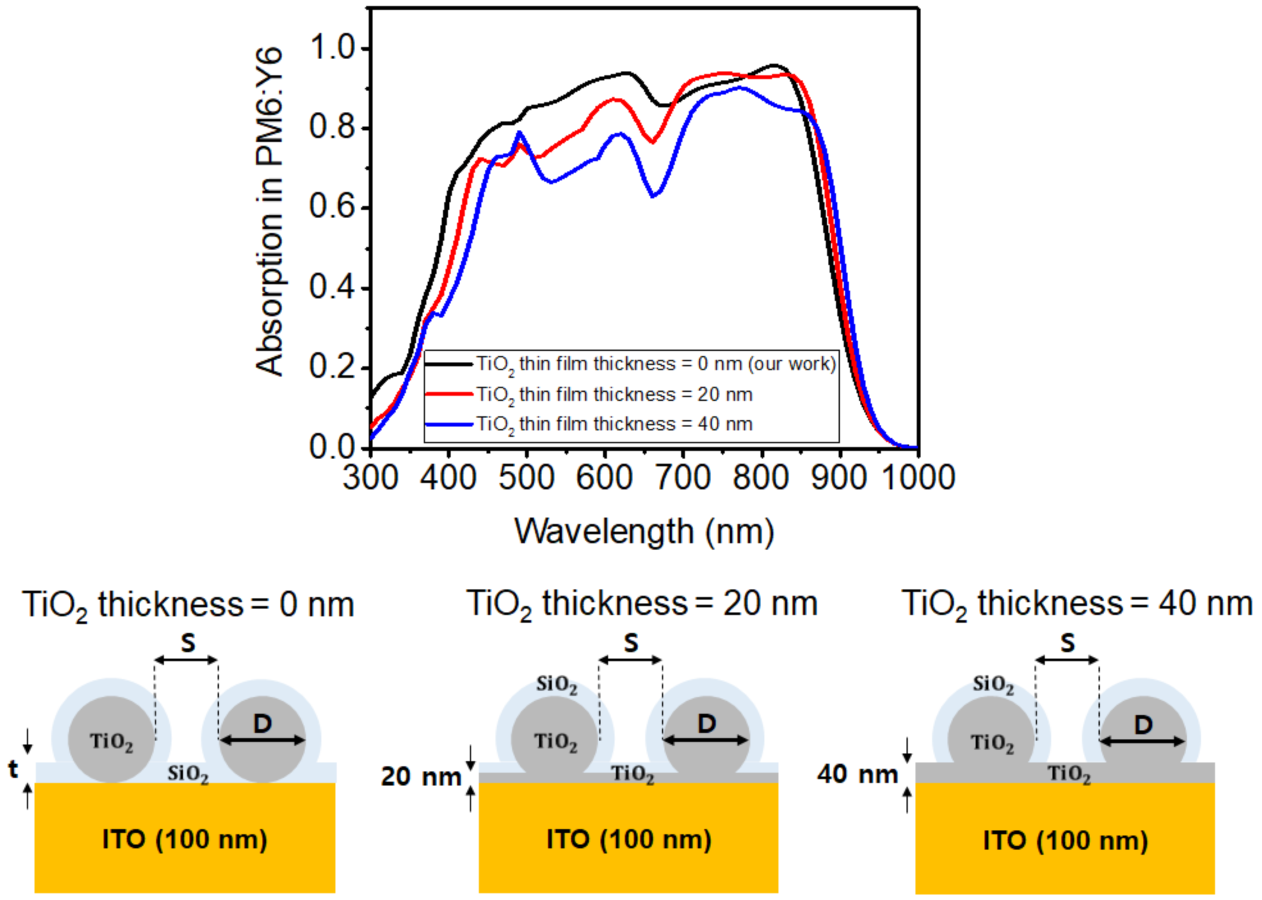

Figure S4. Calculated absorption spectra in the photoactive layer obtained from the proposed structures with 20 nm-thick and 40 nm-thick TiO2 thin films on top of ITO.

References

1. Ratzsch, Stephan, et al. "Influence of the oxygen plasma parameters on the atomic layer deposition of titanium dioxide." *Nanotechnology* **26** 024003 (2014).

2. Gao, L., Lemarchand, F. & Lequime, M. “Refractive index determination of SiO2 layer in the UV/Vis/NIR range: spectrophotometric reverse engineering on single and bi-layer designs.” *JEOS:RP* **8** (2013).
